# Superior mesenteric artery syndrome, an uncommon cause of gastric outlet obstruction: Case report

**DOI:** 10.1016/j.ijscr.2025.111692

**Published:** 2025-07-15

**Authors:** Orujul Hassan, Clarence Sumbizi, Abduel Kitua, Jacqueline Gabone, Zainab Fidaali, Athar Ali

**Affiliations:** a Department of Family Medicine, The Aga Khan University, P.O. Box 38129, Dar Es Salaam, Tanzania; b Department of Surgery, The Aga Khan University, P.O. Box 38129, Dar Es Salaam, Tanzania; c Department of Radiology, The Aga Khan Hospital, P.O. Box 2289, Dar Es Salaam, Tanzania; d Department of Surgery, The Aga Khan Hospital, P.O. Box 2289, Dar Es Salaam, Tanzania

**Keywords:** Superior mesenteric artery syndrome, Gastric outlet obstruction, Duodenojejunostomy, Case report, Delayed diagnosis, Resource-limited settings

## Abstract

**Introduction and importance:**

Superior Mesenteric Artery (SMA) syndrome is an uncommon cause of gastric outlet obstruction secondary to duodenal compression between the SMA and the abdominal aorta. Diagnosis is challenging due to its nonspecific symptoms. We present a case highlighting an atypical presentation in a patient with normal BMI, prolonged diagnostic delay, and surgical management in a resource-limited setting.

**Case presentation:**

We report a case of an 18-year-old female who presented with chronic abdominal pain, nausea, vomiting, and early satiety over three years. Despite a normal BMI (23.8 kg/m^2^), contrast-enhanced CT showed a reduced aortomesenteric angle (17°) and distance (4.5 mm), confirming SMA syndrome. Conservative management failed, necessitating duodenojejunostomy, which led to complete symptom resolution.

**Clinical discussion:**

SMA syndrome is an uncommon and challenging diagnosis, especially in patients without classical presentations such as low BMI. Delayed diagnosis contributed to prolonged morbidity. The successful surgical intervention in a resource-limited setting emphasizes the need for increased awareness of SMA syndrome as a differential for chronic upper GI symptoms.

**Conclusion:**

This case outlines the diagnostic challenges SMA syndrome poses due to its nonspecific nature of the clinical presentation. Improved clinical suspicion can reduce diagnostic delays and optimize management, particularly in regions with limited diagnostic resources.

## Highlights

• Atypical Presentation with Normal BMI – Unlike most reported cases of Superior Mesenteric Artery (SMA) Syndrome, which are associated with severe weight loss and low BMI, this case involved a patient with a normal BMI (23.8 kg/m^2^), challenging conventional diagnostic expectations.
• Prolonged Diagnostic Delay – The patient experienced three years of chronic, nonspecific gastrointestinal symptoms before an accurate diagnosis was made, highlighting the difficulty in recognizing SMA syndrome and the risk of misdiagnosis or delayed intervention.
• Diagnostic Challenges in a Resource-Limited Setting – The case underscores the importance of clinical suspicion and appropriate imaging in settings where advanced diagnostic tools may not be readily available, emphasizing the need for awareness among healthcare providers.
• Successful Surgical Intervention After Failed Conservative Management – Despite an initial trial of conservative therapy (nutritional support, prokinetics, and postural adjustments), symptoms persisted, necessitating duodenojejunostomy, which led to complete symptom resolution.
• First Documented Surgical Case of SMA Syndrome in Tanzania – Based on current literature, this is the only reported case from Tanzania where surgical intervention was performed for SMA syndrome, contributing to global data on the condition and its management in low-resource settings.

## 1 Introduction

Superior mesenteric artery (SMA) syndrome, also known as Wilkie syndrome, cast syndrome, chronic duodenal ileus, or arterio-mesenteric duodenal obstruction [1] is one of the rare causes of gastric outlet obstruction (GOO) with an estimated incidence of about 0.013–0.3 % [2]. It results from a narrowing at the angle of the origin of the superior mesenteric artery from the abdominal aorta (AA) causing a reduction in the aortomesenteric (AOM) distance, eventually leading to obstruction at the 3rd part of the duodenum [3]. The normal aortomesenteric angle ranges between 38° and 65°, with an aortomesenteric distance ranging from 10 mm to 34 mm [4]. The angle is maintained mainly by the mesenteric fat pad. In SMA syndrome, these values decrease to 6°–22° and 2–8 mm, respectively [4].

Multiple risk factors have been outlined that may contribute to the development of SMA syndrome, which may be classified into congenital anomalies or acquired factors that primarily lead to loss of the mesenteric fat pad or trauma that changes the shape of the abdominal wall or spine [5]. Patients with SMA syndrome typically present with nonspecific symptoms including early satiety, nausea, vomiting, postprandial abdominal pain often relieved by positional changes, and progressive weight loss which may worsen the loss of mesenteric fat pad further triggering the narrowing of the AOM angle and distance entering in a vicious cycle [6].

Due to the nonspecific nature of the symptoms, the diagnosis of SMA syndrome is usually difficult and challenging [7]. Diagnosis is primarily radiological, with CT scans revealing duodenal obstruction and a reduced AOM angle [8]. Treatment of SMA syndrome is of significance as it can cause dehydration, malnutrition, metabolic imbalance, and in some cases, death [9]. Initial management typically involves conservative measures through postural adjustments, enteral nutrition, and prokinetics, which has been found to have a success rate of about 70 %–80 % [10,11]. However, surgical intervention is required for refractory cases, with duodenojejunostomy being the preferred procedure [[12], [13], [14], [15]].

We report a case of an 18-year-old female who presented to our facility with chronic vague abdominal symptoms of more than 3 years, who on further radiological workup revealed to have gastric outlet obstruction secondary to superior mesenteric artery syndrome. We describe our experience from a tertiary-level hospital in Dar es Salaam, Tanzania highlighting our approach to an unusual surgical cause of gastric outlet obstruction. This paper has been reported in line with the revised SCARE 2025 criteria [16].

## 2 Case presentation

An 18-year-old female patient with no known comorbidities, and no known drug allergies, presented to our facility with chronic abdominal pain for 3 years, which was more on the epigastric region, gradual in onset, and progressively worsening with time, it was dull in nature, on and off, no specific periodicity, radiating to the para umbilical area occasionally. It was exacerbated after eating with no known relieving factor. It was accompanied with heartburn, nausea, and occasional vomiting, containing recently eaten food content - occasionally bile-stained. She also reported having early satiety and subjective unintentional weight loss manifested by her clothes not fitting her anymore.

She denied any abdominal swelling, abdominal distension, fever, change in stool, or urinary habits. There was no history of cough, night sweats, or any history of any recent TB contact. The patient denied any history of abdominal trauma or any prior surgeries. The patient had no history of scoliosis, spinal surgery, intestinal surgeries, or congenital anomalies. There was no history of foreign body ingestion, no history of alcohol use, or cigarette smoking. She denied any known family history of cancer or any similar presentation. She was sexually inactive, with her last menstrual period 2 weeks before admission. She had visited multiple local dispensaries for chronic pain over the past 3 years for which she was given proton pump inhibitors and painkiller medications with minimal relief of the symptoms. She had never been diagnosed with H.Pylori positive peptic ulcer disease throughout her visits.

On examination, she was alert, oriented, afebrile, and not wasted with a normal body mass index (BMI) of 23.8 kg/m^2^ and had stable vitals. Her abdominal examination revealed a scaphoid abdomen with no visible scars, no swellings, no peristalsis, and was moving with respiration, on palpation had a soft abdomen with mild tenderness in the epigastric area on deep palpation, with no organomegaly and no rebound tenderness, succussion splash test negative, normal tympanic percussion, normal bowel sounds. The rest of the systemic examination was essentially normal.

**Fig. 1:**
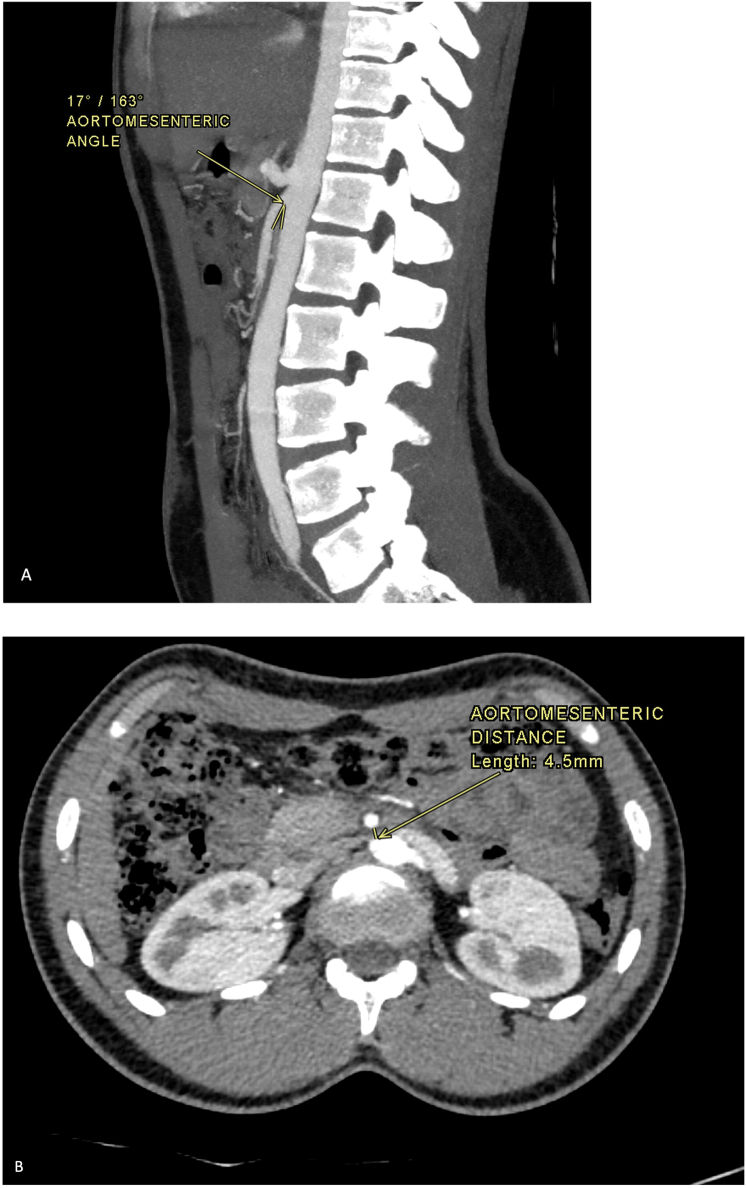
Contrast enhanced CT of the abdomen, sagittal image (A) and axial image (B). Acute angulation of the superior mesenteric artery was identified; The aortomesenteric angle and distance were both reduced measuring measured 17° and 4.5 mm respectively (measurements shown on A and B respectively).

Multiple laboratory investigations were ordered including a complete blood count, electrolytes, complete renal and liver profiles, and pancreatic enzymes which were all unremarkable. A CT scan of the abdomen with contrast was ordered which revealed a mildly distended stomach with normal wall thickness and complete gastric emptying during the study time, a fluid-filled proximal duodenum with tapered narrowing at the 3rd part as it passed between the superior mesenteric artery and descending aorta, acute angulation of superior mesenteric artery with an aortomesenteric angle of 17°, reduced aortomesenteric distance of 4.5 mm, with no evidence of compression of the left renal vein concluding duodenal compression secondary to Superior mesenteric artery syndrome with no evidence of Nutcracker syndrome (Fig. 1).

**Fig. 2:**
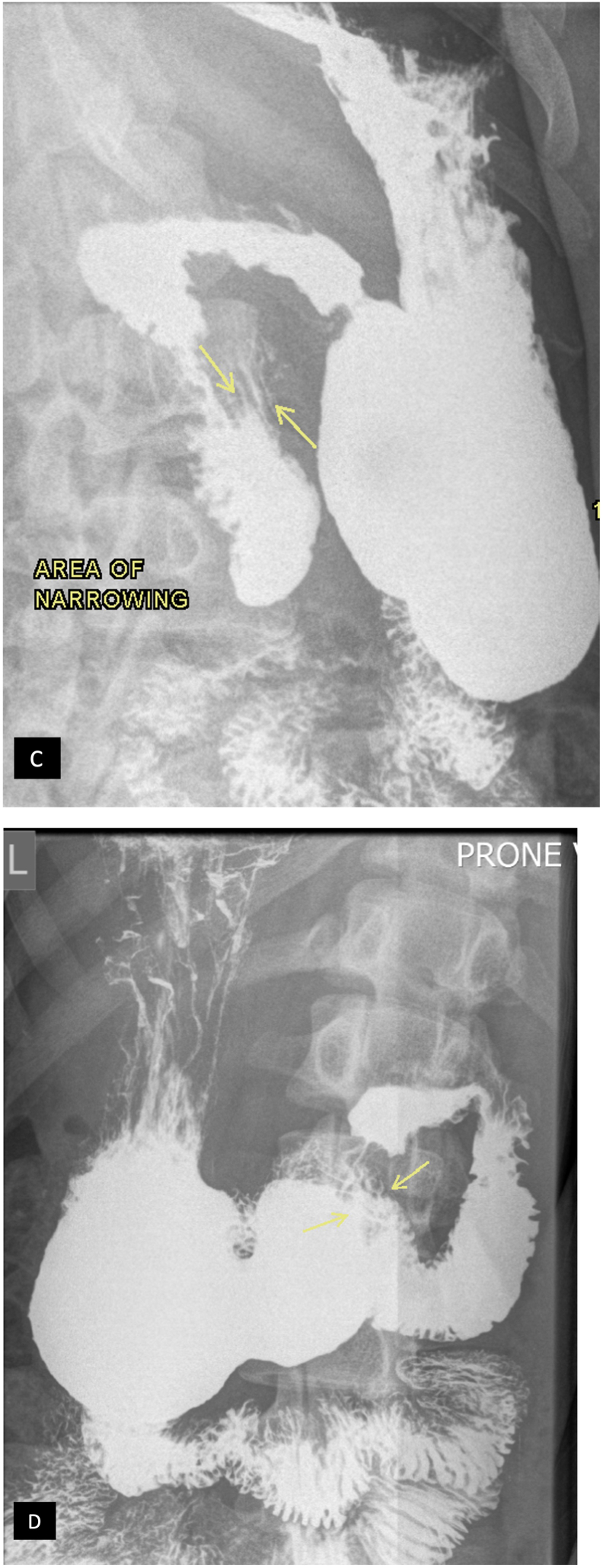
Barium meal follow on erect abdominal radiograph demonstrates in (C) dilated first and second portion of the duodenum with tapered narrowing at the third part of the duodenum (arrows). On repositioning the patient to prone (D), the oral contrast was seen passing into the third and fourth part of duodenum (arrows).

Further barium study was done with a barium meal to elicit the extent of the impact of the angulation on the duodenal lumen which demonstrated minimal passage of contrast fluid at the tapered narrowed 3rd part of the duodenum with increased spastic duodenal peristalsis causing contrast to reflux in the stomach, however on repositioning the patient to prone (increasing the angulation), the contrast was seen to pass suggesting partial gastric outlet obstruction partly relieved by positional changes (Fig. 2).

The patient was then informed of the diagnosis and counselled on the management plan which was initially a conservative approach whilst actively following up the patient by allowing weight gain with nutritional supplementation through frequent, high-caloric small meals and engaging in posture therapy when experiencing symptomatic pain. An upper GI endoscopy (EGD) was considered but not performed, as the patient's symptoms and imaging findings strongly supported a mechanical extrinsic obstruction consistent with SMA syndrome. There were no signs suggestive of mucosal lesions such as bleeding or ulceration. A proton pump inhibitor (Rabeprazole 20 mg 12hourly) and a prokinetic (Domperidone 10 mg 12hourly) were given to relieve symptoms temporarily.

Upon actively following up with the patient weekly, until the 3rd week, there was minimal improvement in symptoms and weight gain. After an extensive conversation with the patient and her family, the decision was made by the patient for surgery rather than conservative management and was prepared for an explorative laparotomy with duodenojejunostomy bypass.

**Fig. 3:**
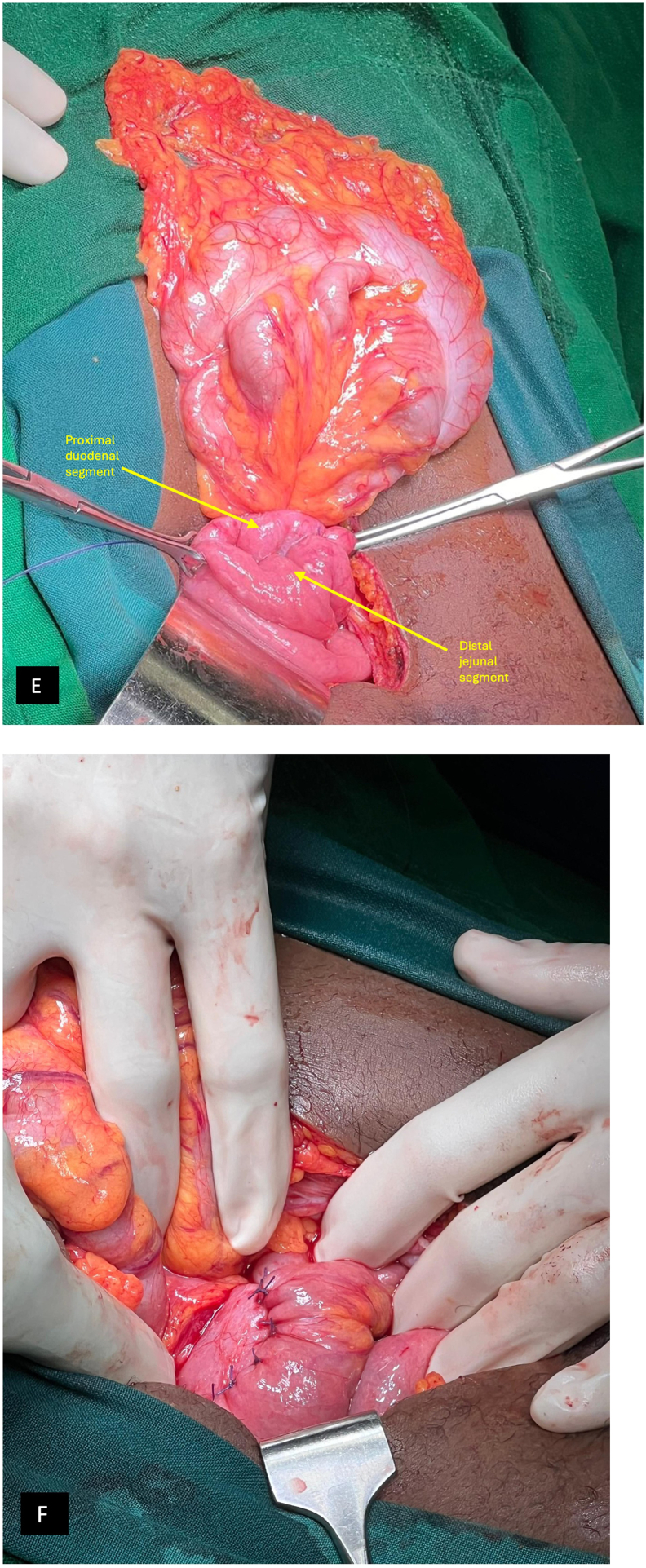
Duodenojejunostomy. (E) Distal duodenal loop placed side to side with proximal jejunal loop bypassing the segment compressed by Ligament of Treitz (F) Side to side anastomosis between proximal duodenum and distal jejunum.

Intraoperative findings revealed significant compression of the third part of the duodenum by the ligament of superior mesenteric artery, with a markedly narrowed aortomesenteric angle. The proximal duodenum and stomach were distended, while the jejunum distal to the ligament of Treitz was minimally collapsed but demonstrated a patent lumen. Manual gastric decompression was performed via the nasogastric tube on suction, followed by mobilization of the third part of the duodenum by kocherization through superior mobilization of the transverse colon to gain better access to the descending and horizontal duodenum and bypass of the compressed segment was achieved via a stapled antecolic side-to-side duodenojejunostomy chosen for convenience, although studies show no superiority of the technique as compared to hand-sewn anastomosis in terms of initiation of feeds and risk of anastomotic failure. The anastomosis was tested for adequate patency and leak using 200 mL of normal saline through a nasogastric feeding tube, confirming normal flow without leakage (Fig. 3).

Postoperative care was provided in accordance with the Enhanced Recovery after Surgery (ERAS) protocol [17]. The patient resumed oral intake 24 h postoperatively, starting with ice cubes, progressing to clear liquids, and gradually transitioning to a regular soft diet. She received parenteral analgesics, began ambulation with the assistance of a physiotherapist within 24 h, and had the nasogastric tube (NGT) for decompression removed at 24 h. The postoperative course was uneventful, and she was discharged on day 5 with a regular diet, normal bowel habits, and prescriptions for oral analgesics and proton pump inhibitors. She was scheduled for a follow-up in the outpatient clinic. At discharge, the patient was advised to maintain a high-calorie, nutrient-dense diet to support further weight gain and prevent recurrence. She was instructed to monitor for any recurrence of gastrointestinal symptoms such as abdominal pain, early satiety, or vomiting.

She was followed up at 1 week and again at 1 and 3 months post-discharge. At the most recent visit, she had demonstrated weight gain, with no reported dyspeptic symptoms, fever, abdominal pain, distension, nausea, or vomiting. No other complications were noted, and she had resumed her normal daily activities. She is currently scheduled for her next 6 month follow-up appointment.

## 3 Discussion

SMA syndrome remains underdiagnosed due to its vague presentation and poses a diagnostic challenge [7], as illustrated by our case whereby the patient was symptomatic for more than 3 years before the diagnosis was made. Mandarry et al. [2] emphasized that SMA syndrome should be considered in patients with nonspecific signs and symptoms of upper gastrointestinal tract obstruction.

In our case, the factor to note was the normal BMI despite the patient reporting subjective weight loss, which was atypical in comparison to other studies [[18], [19], [20]] whereby low BMI was a risk factor and most patients were underweight.

The prolonged duration of symptoms before definitive imaging prolonged morbidity and underscores the importance of early recognition [21]. Proning the patient improved the flow of the duodenal content as demonstrated by the barium meal which was in line with Pottorf et al. [12] as positioning such as the left lateral decubitus or prone position releases the aortomesenteric angle providing partial relief of the symptoms.

Conservative management is considered to be the mainstay treatment option for uncomplicated cases [22] but in complicated cases and vicious cycle of this condition, some cases respond only to surgical intervention. The timing and indications of conversion from conservative to surgical approach remains unclear [10]. Current surgical techniques aim to either bypass the obstruction or relieve the compression of the duodenum.

Our patient underwent a duodenojejunostomy which is currently considered the standard of care in SMA syndrome, demonstrating a success rate exceeding 90 % over a 5-year follow-up period [2]. Currently with modern advances in minimally invasive surgery, the laparoscopic technique for duodenojejunostomy has demonstrated safety, effectiveness, shorter hospital stay as well as low morbidity making it a favorable approach [12,14,15,22], however due to limited experience, a more traditional laparotomy approach was used in this case.

To date, this is the only documented reported case of a surgically managed superior mesenteric artery syndrome in Tanzania based on the currently available literature. Most case reports and studies about SMA Syndrome focus on its occurrence in various global regions, often highlighting its rarity and diagnostic challenges. Through our experience, we would like to emphasize consideration of SMA syndrome as a differential in patients with nonspecific signs and symptoms of proximal GI tract obstruction, especially in regions where cases are more likely to be underreported due to limited diagnostic resources .

## 4 Conclusion

In conclusion, superior mesenteric artery (SMA) syndrome is a rare but significant cause of gastric outlet obstruction, requiring a high index of suspicion for diagnosis due to its nonspecific symptoms and diagnostic challenges. While conservative management remains the first line of treatment, surgical intervention provides an effective solution for refractory cases, with excellent outcomes. Increased awareness and reporting of SMA syndrome are crucial to improving diagnosis and management, especially in resource-limited countries where underreporting is common.

## Abbreviations

AA: Abdominal Aorta
AOM: Aortomesenteric
BMI: Body mass index
CT: Computed tomography
ERAS: Enhanced recovery after surgery
GI: Gastrointestinal
GOO: Gastric outlet obstruction
mm: Millimeters
NGT: Nasogastric tube
NPO: Nil per oral
SMA: Superior mesenteric artery
TB: Tuberculosis

## Consent for publication

Written informed consent was obtained from the patient, preserved and ready to be submitted with editor's request.

## Ethical approval

This case report was exempt from institutional review board (IRB) review as per the policies of our institution, which does not require ethics approval for single-patient case reports not constituting human subject research.

## Funding

No funds were needed to publish this case.

## Author contribution

OH: Study conception, production of initial manuscript, collection of data.

C·S: Introduction and discussion.

A.K: Revision of the manuscript, proofreading.

J.G: Clinical radiologist involved in imaging review and radiological reporting.

Z.F: Clinical radiologist involved in imaging review and radiological reporting.

A.A: Study conception, revision of manuscript, proofreading and supervision.

## Guarantor

Dr. Orujul Hassan.

## Research registration number

N/A.

## Declaration of Generative AI and AI-assisted technologies in the writing process

Grammarly (Premium version, accessed December 2024) was used during the writing and revision stages of this manuscript to assist with grammar correction, sentence clarity, and style refinement. No clinical data, patient identifiers, or images were shared, and all content reviewed was fully anonymized and compliant with GDPR/HIPAA regulations. The authors reviewed and approved all suggestions made by the tool and take full responsibility for the accuracy and integrity of the final manuscript. Grammarly was not used for data analysis, clinical interpretation, or figure generation.

## Conflict of interest statement

The authors declare that they have no competing interests.

## Data Availability

The datasets of the present study are available from the corresponding author upon request.
